# Characterization of Mechanical Property Distributions on Tablet Surfaces

**DOI:** 10.3390/pharmaceutics10040184

**Published:** 2018-10-12

**Authors:** Ramon Cabiscol, Jan Henrik Finke, Harald Zetzener, Arno Kwade

**Affiliations:** 1 Institute for Particle Technology (iPAT), Technische Universität Braunschweig, 38104 Braunschweig, Germany; jan.finke@tu-braunschweig.de (J.H.F.); h.zetzener@tu-braunschweig.de (H.Z.); a.kwade@tu-braunschweig.de (A.K.); 2 Center of Pharmaceutical Engineering (PVZ), Technische Universität Braunschweig, 38106 Braunschweig, Germany

**Keywords:** indentation, tablets, inhomogeneity, BET, porosity

## Abstract

Powder densification through uniaxial compaction is governed by a number of simultaneous processes taking place on a reduced time as the result of the stress gradients within the packing, as well as the frictional and adhesive forces between the powder and the die walls. As a result of that, a density and stiffness anisotropy is developed across the axial and radial directions. In this study, microindentation has been applied to assess and quantify the variation of the module of elasticity ($E_{mod}$) throughout the surface of cylindrical tablets. A representative set of deformation behaviors was analyzed by pharmaceutical excipients ranging from soft/plastic behavior (microcrystalline cellulose) over medium (lactose) to hard/brittle behavior (calcium phosphate) for different compaction pressures. The results of the local stiffness distribution over tablet faces depicted a linear and directly proportional tendency between a solid fraction and $E_{mod}$ for the upper and lower faces, as well as remarkable stiffness anisotropy between the axial and radial directions of compaction. The highest extent of the stiffness anisotropy that was found for ductile grades of microcrystalline cellulose (MCC) in comparison with brittle powders has been attributed to the dual phenomena of overall elastic recovery and Poisson’s effect on the relaxation kinetics. As a reinforcement of this analysis, the evolution of the specific surface area elucidated the respective densification mechanism and its implementations toward anisotropy. For ductile excipients, the increase in the contact surface area as well as the reduction and closing of interstitial pores explain the reduction of surface area with increasing compaction pressure. For brittle powders, densification evolves through fragmentation and the subsequent filling of voids.

## 1. Introduction

Throughout the last decades, many techniques have been applied in order to characterize the quality of pharmaceutical tablets. The available set of existing methods ranges from destructive to non-destructive techniques such as ultrasonic pulsing [1], X-ray tomography [2], or terahertz pulsing [3]. Although in its essence microindentation is a technique that provides a depiction of the superficial elasto-plastic behavior of large specimens such as tablets, it is a very valued method due to the reduced requirements of sample preparation and the versatility of usage. Mechanical properties such as elastic modulus or hardness have a strong potential in the quality assessment of the compaction of novel formulations, as well as elucidating the interplay of process parameters (among others, compaction speed, maximum stress, or degree of lubrication) with the product’s properties.

During the compaction of powders, the synergetic combination of mechanical interlocking, elasto-plastic deformation, and fragmentation conveys the final shape and mechanical strength of the tablet [4]. The role of particle morphology and the mechanical properties of the tablet’s constituents have been repeatedly studied in order to estimate the final tensile strength [5,6,7]. The early work of Nyström already set the idea that the tensile strength of tablets in the axial direction was lower than in the radial direction [6,8], which is a behavior that is referred to from this point as “strength anisotropy”. At the same time, Markl et al. suggested that pores are preferably orientated perpendicular to the compaction direction, and that this phenomenon decreases with an increasing solid fraction (SF) [3]. A deeper analysis of the effect of particle morphology on the strength anisotropy of the tablets revealed a higher anisotropic tendency of needle-shaped excipients (acicular) than equiaxed crystals [9]. The evidence of the preferential orientation of acicular grades of microcrystalline cellulose (MCC) lays on the tendency to orient normal to the die axis during filling and tapping. In spite of that, the main contribution to the strength anisotropy is not associated with the morphology of primary constituents, but rather with an uneven confinement due to the higher number of contacts in the normal direction of the tablet. Similar results were achieved by investigating the uniaxial compression of solid balls at lower stresses [10].

As a result of the confinement of the powder during compaction, friction and adhesion between the powder and the die walls also play a crucial role in the quality of the final tablet. However, an excessive die wall friction can induce a shear force that results in irregular density distributions within the tablet [11]. A lack of porosity uniformity can be an indicator of regions of differing stored energy, which may convey into the occurrence of defects during finishing operations (capping and lamination, for instance) and even poor product performance [2]. 

Indentation has also been applied in pharmaceutics for the assessment of quality standards in small batch production [12]. One of the main criticisms toward this technique is the low spatial resolution because of the low number of sampling points. At the same time, a big challenge that is associated with the application of the indentation on pharmaceutical tablets for the generation of global results are structural inhomogeneities. Additionally, the speed-dependent deformation of compacts makes it tedious to identify repeatable measuring parameters [13]. However, microindentation is a reliable, comparable, and straightforward method, and it constitutes a good complement of optical analytical tools (such as near-infrared (NIR) or photoacoustics [14,15,16]). In the latter case, challenging calibration trials need to be carried out in order to correlate the measured information with structural parameters.

In case of the nanoindentation and microindentation of flat specimens, a wide set of different tip geometries has been developed [17]. Some reports have presented the indentation of tablets with sharp or spherical indenters. Ridgway et al. and Wenzel et al., for instance, determined the microindentation hardness of acetylsalicylic acid and MCC tablets, respectively, using a Vickers indenter [18,19]. Both authors found that for tablets produced on an eccentric lab press, the hardness of the upper side of the tablets is always higher than the lower face. Wenzel went one step beyond and identified the flipping of the upper face at high compaction pressures: the upper surface became softer than the lower surface as a consequence of the elastic recovery. In parallel, Jetzel and Leuenberger set a numerical relationship between Brinell hardness, compaction pressure, and tablet porosity, making hardness a tool to predict the compressibility and compactibility of final tablets [20,21].

A particular challenge of indentation with a flat-end cylinder is that few reports of the applicability and validity of this approach can be found, most of them coming from metallurgy [22,23]. The main characteristic of this approach is that the contact area remains constant as the load increases. Yu et al. developed an indentation model for different shapes of indenters based on the elastic theory by Hertz [24]. By their assumption, specimens are considered linearly elastic and indenters are considered rigid bodies; thus, the theory of elasticity applies. This model is only applicable to materials with a low ratio of elastic modulus to the yield stress. Two main conditions restrict the applicability of this model: the first imposes that the free surface outside the contact region has no normal stress acting on it; the second assures the absence of any friction for the contact region between the indenter and the half space [22]. 

The purpose of the current work is to depict the anisotropy of the mechanical properties between the radial and axial parts, as well as the radial dependency of the stiffness of single-component tablets for different process parameters. A representative set of deformation behaviors was analyzed by compacting the common pharmaceutical excipients, ranging from soft/plastic behavior (MCC) over medium (lactose) to hard/brittle behavior (calcium phosphate). They are employed to depict the influence of compaction pressure on the stiffness distribution in tablets.

In addition to that, this study correlates those heterogeneities derived from microindentation results with meaningful parameters of the arrangement and deformation behavior of the constituents, such as the elastic recovery and the specific overall surface area of the tablets. The specific surface area, as determined by experimental isotherm equation of Brunauer, Emmett, and Teller (BET), is evaluated for all of the formulations to explain how the evolution of specific surface area is associated with the degree of consolidation of the sample and the mechanical behavior of each excipient.

## 2. Materials and Methods

### 2.1. Materials

Four different excipient grades are used for tableting: Avicel^®^ PH 200 (FMC BioPolymer, Philadelphia, Pa, USA) (MCC-A), Pharmacel^®^ 102 (DFE Pharma, Nörten-Hardenberg, Germany) (MCC-P), Tablettose^®^ 100 (Meggle Pharm, Wasserburg am Inn, Germany) (LAC), and DI-CAFOS^®^ D160 (Budenheim, Budenheim, Germany) (CAPH). The first two constitute two grades of microcrystalline cellulose with a different particle size distribution: Tablettose^®^ 100 is a lactose monohydrate, whereas the main component of DI-CAFOS^®^ D160 is dicalcium phosphate dihydrate.

### 2.2. Powder Characterization

Particle size distribution (PSD) and the particle morphology of MCC-A/P and CAPH are determined via dynamic image analysis (QICPIC, Sympatec GmbH, Clausthal-Zellerfeld, Germany) equipped with a vibrating chute to disperse particles into a free-fall funnel (GRADIS, Sympatec GmbH, Clausthal-Zellerfeld, Germany). Due to the cohesive behavior of LAC, its PSD is determined by laser diffraction with dry dispersion via airflow injector with a dispersion pressure of 2 bar (HELOS laser diffraction, RODOS dispersion system, Sympatec GmbH, Clausthal-Zellerfeld, Germany).

In order to get deeper information about the aspect, shape, and morphology of the analyzed materials, scanning electron microscope (SEM) acquisitions are also presented. Materials were sputtered with gold and investigated with a field emission SEM (LEO 1530, Carl Zeiss Microscopy GmbH, Jena, Germany), applying an acceleration voltage of 5 kV and a working distance of 10 mm. Different magnifications between 150× and 3000× were applied.

The true or skeletal density is determined by means of helium pycnometry (ULTRAPYC 1200e, Quantachrome GmbH, Odelzhausen, Germany). Samples were stored at 20 °C and 45% RH during the 24 h prior to the analysis. Measured values are extracted after 10 runs of the same sample and assuring a typical deviation lower than 0.5%.

### 2.3. Tableting

Each formulation is compacted using the compaction simulator STYL’One Evolution (Medel Pharm S.A.S., Beynost, France). Standard EURO B die and punches were installed in order to produce cylindrical tablets that were 11.28 mm in diameter. The compaction sequence comprises the filling of the die by a gravity fill shoe up to a height of 10 mm with the powder of interest (conditioned for all cases at 20 °C and 45% RH for 24 h), and the symmetrical movement of the punches at a constant speed of 20.6 mm/s until the target pressure is achieved.

Due to the poor processability of pure Tablettose^®^ 100 and DI-CAFOS^®^ D160, each of these batches is internally lubricated by blending 1% (*w/w*) of magnesium stearate (MgST) (Magnesia 4264; Magnesia GmbH, Lüneburg, Germany) in a powder mixer ERWEKA AR-403/-S (ERWEKA GmbH, Heusenstamm, Germany) with a 3.5-L cubic container for 3 min at 16 rpm before tableting. All four grades of powder freely flowed from the feeder to the die, precisely limiting the fill volume by the position of the lower punch. The excess of powder was scraped off the die, yielding reproducible fill and tablets with constant weights for the same processed batch.

In the current study, compaction pressures running from 30 MPa up to 250 MPa are investigated.

In order to examine the relaxation phase after compaction, three magnitudes of elastic recovery are calculated. In-die axial elastic recovery ($ER_{die-ax}$) represents the immediate elastic recovery expressed as:

(1) $$ER_{die-ax}=\frac{PD_{0}-PD_{min}}{PD_{min}},$$

where $PD_{min}$ is the distance between the upper and the lower punch at the target compaction pressure, and $PD_{0}$ is the distance at the point of contact force = 0 during the withdrawal of the upper punch.

The axial 24-h elastic recovery ($ER_{24-ax}$) is extracted from the nominal height of the tablet after a relaxation period of 24 h, and it is defined as:

(2) $$ER_{24-ax}=\frac{h_{24}-PD_{min}}{PD_{min}},$$

where $h_{24}$ is the height of the tablet after a relaxation period of 24 h after the compaction. Finally, the radial relaxation of the tablet is analyzed by the 24 h radial elastic recovery ($ER_{24-rad}$) as:

(3) $$ER_{24-rad}=\frac{{Ø}_{24}-{Ø}_{imm}}{{Ø}_{imm}},$$

where ${Ø}_{24}$ is the tablet diameter after a relaxation period of 24 h, and ${Ø}_{imm}$ is the die diameter.

### 2.4. Tablet Characterization

The attributes and properties of final tablets are extracted 24 h posterior to the compaction. Diameter (${Ø}_{24}$), height ($h_{24}$), and breakage force ($F_{bre,\;24}$) are determined by the MultiTest 50 (Dr. Schleuniger Pharmatron, SOTAX AG, Allschwil, Switzerland). $F_{bre,\;24}$ is measured by diametrical compression, applying a constant speed of 0.35 mm/s. The tensile strength ($\sigma_{t}$; a critical mechanical property for transportation, finishing, and handling [25]) is determined for flat-faced compacts as:

(4) $$\sigma_{t}=\frac{2F_{bre,\;24}}{\pi {Ø}_{24}h_{24}},$$

The density–pressure relationship developed at the early work of Heckel [26] is widely used to analyze the deformation of powders during compaction with pressure:

(5) $$ln\left(\frac{1}{\varepsilon }\right)=kP+A,$$

The parameters P and A account for the compaction pressure and particle rearrangement at low pressures, respectively [27]. Previous analysis identified the linear range of Heckel data [28,29]. Although experimental data is never truly linear, it can be considered so at low pressure intervals (50–100 MPa). Therefore, linear regression is used to extract the average slope for each material at the pressure range of interest. The inverse of this slope (k) constitutes the so-called yield pressure ($P_{y}$), which is used to classify the deformation behavior of the materials. According to the classification defined by Führer et al., materials with $P_{y}$ < 80 MPa are defined as soft/plastic, whereas above this limit, excipients are expected to exhibit a hard/brittle behavior [30].

The specific surface area of tablets is determined by nitrogen sorption using the NOVA 2000e Surface Area and Pore Size Analyzer (Quantachrome GmbH, Odelzhausen, Germany). After compaction, cylindrical tablets are broken into quarters and inserted into the degassing chambers, where they are treated for 24 h at room temperature at vacuum conditions in order to remove physisorbed compounds. Then, a conditioning interval of 500 s precedes the sorption of nitrogen at a pressure ratio with increasing relative pressure levels of 0.10, 0.15, 0.20, 0.25, and 0.30, and an absolute temperature of −196 °C. Samples are measured in quadruplicate. The specific surface area is calculated with the best linear fit for the BET model.

### 2.5. Microindentation

The Universal Nanomechanical Tester (UNAT; ASMEC, Advanced Surface Mechanics GmbH, Dresden, Germany) is employed for obtaining the microindentation data. Tests are performed at room temperature after storing the samples at 20 °C and 45% relative humidity for 24 h prior to the test beginning. 

**Figure 1:**
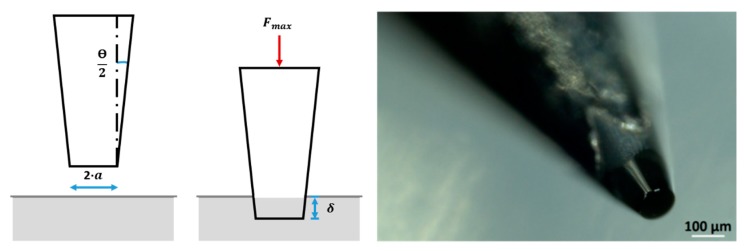
(**Left**) Schematic view of indentation with a frustum-shaped indenter with an opening angle θ = 40° and a punch radius a = 50 µm. $F_{max}$ corresponds to the maximum indentation force and δ, the indentation depth at this force; (**Right**) Microscope acquisition of the 100 µm flat punch.

Given the goal to provide a wide resolution of indents over the tablet faces without compromising the tablet structure, UNAT is assembled with a 100-µm diameter frustum-shaped flat punch (cut cone) (Figure 1) with an opening angle (θ) of 40°. The selection of this indenter size is based on establishing a compromise between a good indentation surface resolution and maximizing the surface area of each indent, not interfering with the morphology for adjacent indents. A common phenomenon in stiffness measurements is the indentation size effect. Briefly, this can be described as an increase in hardness as the indentation size or load is reduced [31]. The phenomena of the indentation size effect is considered not relevant for a total indentation load and an indentation area around 10 MPa and 0.02 mm^2^, respectively.

**Figure 2:**
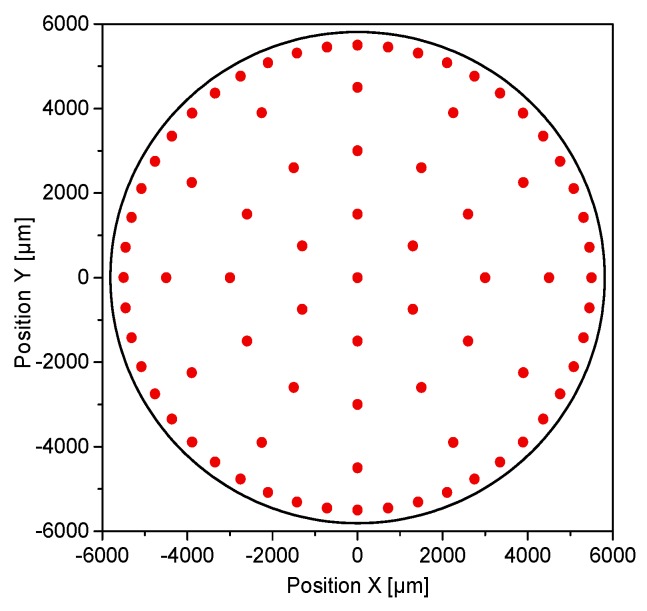
Relative position of the indentation locations throughout the flat face of the cylindrical tablets.

Stiffness distribution is assessed at 166 different locations throughout the upper (79), lower (79), and lateral surface (8) of the tablet. Tablets are glued with a two-sided adhesive tape onto a steel substrate plate. The absolute coordinates of the tablet center [X, Y] are determined with a laser pointer coupled to a microscope. The positions of the 79 indents over the top and the bottom surfaces are imported via a relative coordinates map into the device’s software, and the sequence is fully automatized (Figure 2). In order to perform indents on the lateral surfaces, tablets are placed vertically on their lateral face, held between two steel plates, and glued to the substrate plate. The location of each individual indent is manually controlled and executed on the highest position of the lateral surface, where the indentation area is as planar as possible. 

To provide sufficient statistics, measurements on each surface are performed on eight individual tablets, and no tablet is reused for subsequent tests on other faces. Adjacent indentation points are taken with a distance between centers of at least 700 µm (600 µm between indentation perimeters). A sensitivity analysis of the effect of the perimetrical distance between adjacent indents for a Berkovich punch on WC–Co alloys estimated that a separation of barely 15% of the diametrical indent size would not influence the results [31]. Although the current test case is drastically different than the previous report, a separation threshold of 600% of the primary indent size is considered to be sufficient.

Assuming that the elastic theory is applicable for this scenario, for a given penetration depth h, the mean contact pressure $p_{m}$ is expressed as:

(6) $$p_{m}=\frac{2E_{mod}h}{\pi a\left(1-\nu^{2}\right)},$$

where $E_{mod}$, is the elastic modulus, a is the radius of the punch, and ν is the Poisson’s ratio of the specimen [22]. To solely determine the elastic term of the applied pressure, and exclude the share of plastic deformation taking place during loading, only the unloading recovery height $h_{ela}$ should be considered for this term. Thus, using the mean force at a maximum indentation depth ($F_{max}$) instead of contact pressure and reorganizing Equation (6), $E_{mod}$ can be determined as:

(7) $$E_{mod}=\frac{F_{max}\left(1-\nu^{2}\right)}{2ah_{ela}},$$

**Figure 3:**
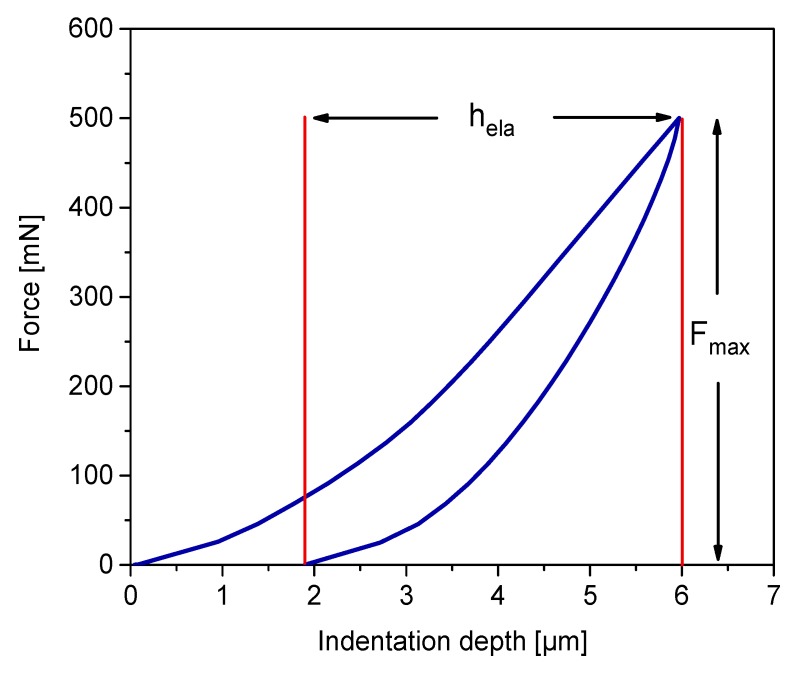
Example of force–displacement curve on a tablet. Material: Pharmacel^®^ 102. Compaction pressure: 175 MPa. Max. indentation force: 500 mN.

The Poisson’s ratio is assumed to be 0.3 for all of the materials [32,33]. Current indentation analyses are performed with maximum force control, which means that the indenter goes into the sample at a constant force rate until it reaches the predefined maximum force target; then, it travels back until complete withdrawal. Taking the typical profile of an indentation curve (Figure 3), the elastic modulus of a compact can be extracted from the maximum indentation force and the unloading recovery height $h_{ela}$.

## 3. Results and Discussion

### 3.1. Powder Characterization

**Figure 4:**
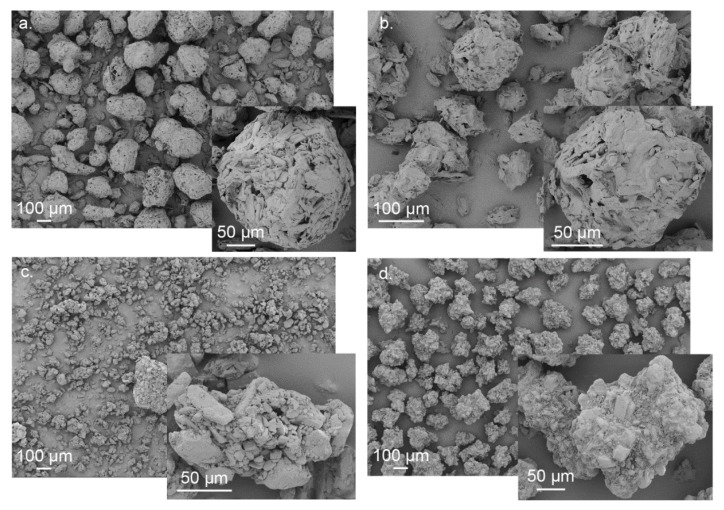
SEM acquisitions of the excipient powders prior to compaction: (**a**) MCC-A; (**b**) MCC-P; (**c**) LAC; (**d**) CAPH. MCC-A: Avicel^®^ PH 200 (FMC BioPolymer, Philadelphia, PA, USA); MCC-P: Pharmacel^®^ 102 (DFE Pharma, Hardenberg, Germany); LAC: Tablettose^®^ 100 (Meggle Pharm, Wasserburg am Inn, Germany); CAPH: DI-CAFOS^®^ D160 (Budenheim, Budenheim, Germany).

From SEM imaging, the morphology of both MCC powders corresponds to a quasi-spherical, equiaxed structure without the presence of individual fibers or fine fragments (Figure 4). These structures consist of aggregates formed from elongated to fibrous primary particles. The occurrence of some slight differences in morphology and size between grades results from different manufacturing conditions. In contrast to that, CAPH and LAC present a quasi-equiaxed structure with substantial morphology differences in comparison with MCC grades. Each aggregate particle consists of smaller, less elongated, crystalline primary particles. In the case of LAC, the presence of prominent agglomerates or clumped structures has been identified [34]. 

The particle size distribution and other physical characteristics of all four materials are summarized in Table 1. It can be seen that MCC-A and CAPH possess x_50_ values in the range comprised between 200–250 µm, while MCC-P and LAC are also comparable in size, but ranging from 80–110 µm, which is approximately half the size of the former. The polydispersity of both MCC grades is comparable, according to their span values, whereas CAPH and LAC constitute two opposite examples of PSD broadness: 0.79 for CAPH and 2.48 for LAC. 

### 3.2. Tablet Quality Parameters: Tensile Strength, In-Die Elastic Recovery and 24 h Elastic Recovery

**Figure 5:**
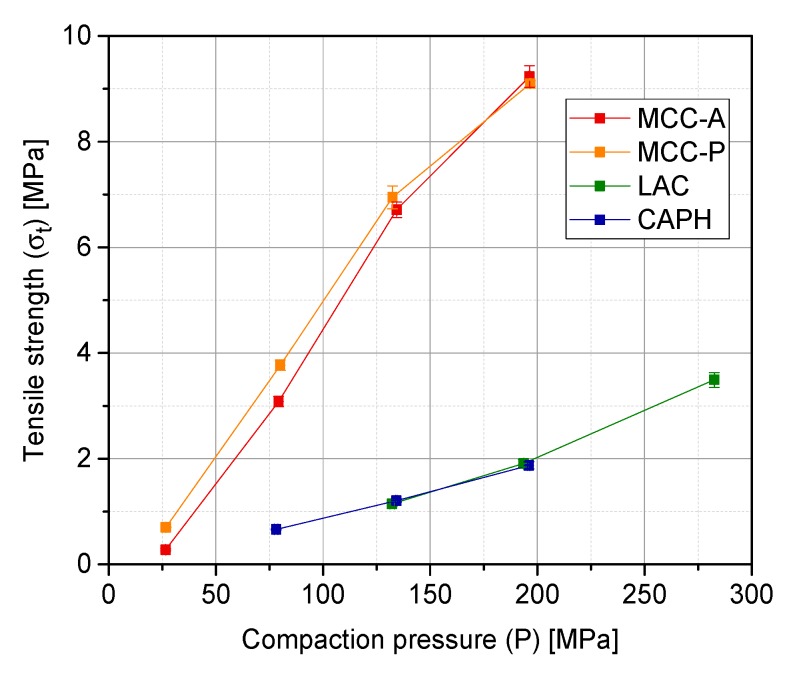
Tensile strength of MCC-A/P, LAC, and CAPH tablets (*n* = 6).

Figure 5 summarizes the evolution of tensile strength with compaction pressure. Two different behavior tendencies are observed: both MCC grades form strong tablets with a slightly higher strength for the finest grade, whereas materials with a more brittle behavior (LAC and CAPH) lead to structures that are roughly four times less strong at the same compaction pressure. Although the internal admixing of magnesium stearate dramatically improves the flowability of the powders from the filling shoe to the compaction die, it has a negative effect on the hardness and tensile strength of the tablets. Tablet strength depends on the area of intimate contact between particles and the attractive forces over the entire contacting area. The fine lubricant particles can interfere with the interactive bonding forces between the particles to be compressed, thus reducing the eventual strength of the tablets [35]. 

**Figure 6:**
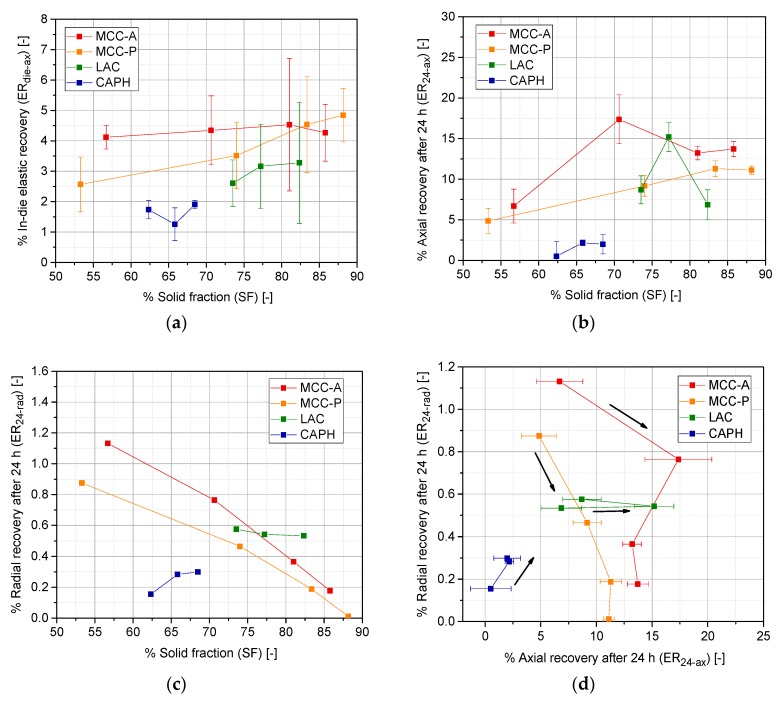
Elastic recovery after compaction of MCC-A/P, LAC, and CAPH granules: (**a**) In-die axial elastic recovery; (**b**) Axial recovery after 24 h; (**c**) Radial recovery after 24 h; (**d**) Axial vs. radial elastic recovery after 24 h (arrows pointing towards the direction of increasing solid fraction).

In-die or quick elastic recovery ($ER_{die-ax}$), 24-h axial elastic recovery ($ER_{24-ax}$), and 24-h radial elastic recovery ($ER_{24-rad}$) are represented in Figure 6. On one hand, both MCC grades exhibit a higher extent of axial elastic recovery, compared with LAC or CAPH (Figure 6a,b), regardless of the degree of densification. This is attributed to the elasto-plastic densification mechanism of both MCC grades, and it constitutes a behavior that is expected beforehand. On the other hand, Figure 6c,d illustrates a drastic decrease of $ER_{24-rad}$ for the highest solid fraction of MCC, approaching zero from a solid fraction of 0.87. However, brittle materials do not exhibit this drastic drop in $ER_{24-rad}$, suggesting that for MCC, the dual competence of overall elastic recovery and Poisson’s effect determines the relaxation kinetics of this type of powder.

Elastic recovery integrates the time-dependent elastic expansion of the final tablet, and it is meant to take place in all possible directions [36,37]. Poisson’s effect is related with the viscoelastic recovery of elasto-plastic excipients [38,39]. At the first instance of compaction, when a uniaxial force is applied within the die, a normal force vector axially presses the granules, which tend to expand toward the perpendicular direction. Due to the in-die confinement, free interstices rapidly shrink at the intermediate stages of compaction, causing a range of two-crossed effects according to the mechanical behavior of the primary particles in a later stage of compaction: particle fragmentation for brittle materials or plastic deformation causing large interfacial areas between particles for ductile materials. In the case of MCC, as a consequence of the aforementioned Poisson’s effect, particles tend to recover their original form after compaction, provoking an axial expansion and a reduction of the radial dimension of the tablet (Figure 6d). However, due to the higher confinement at high solid fractions, Poisson’s effect is no longer possible and therefore, overall, the relative radial elastic recovery becomes almost inexistent. What contributes the most to the overall densification of brittle materials is fragmentation and the rearrangement of the fragments. In this case, elasto-plastic deformation is restricted to the very initial stages of compaction. Therefore, $ER_{die-ax}$ and $ER_{24-ax}$ remain almost constant regardless of the compaction pressure and the resulting solid fraction.

### 3.3. Evaluation of Densification Mechanism

A previous study of compaction mechanisms by the analysis of Heckel plots classified these four excipient grades into two distinct groups [40]. On the one hand, MCC grades with a yield pressure ($P_{y}$) below 50 MPa can be identified as highly plastic and soft materials. On the other hand, LAC (=92.2 MPa) belongs to the range of hard/brittle materials; consideration is attributed to its high crystallinity, which gives it a higher cracking propensity. Finally, for calcium phosphate, although tentative tests located it below the yield limit (=70.1 MPa), the processability of this excipient conveyed the opposite idea: it has to be categorized as a hard elastic material.

**Figure 7:**
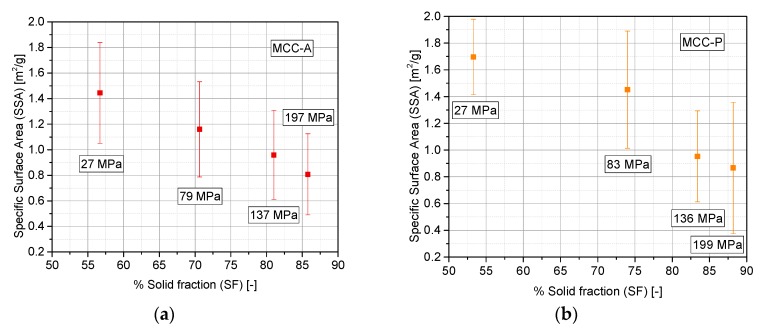

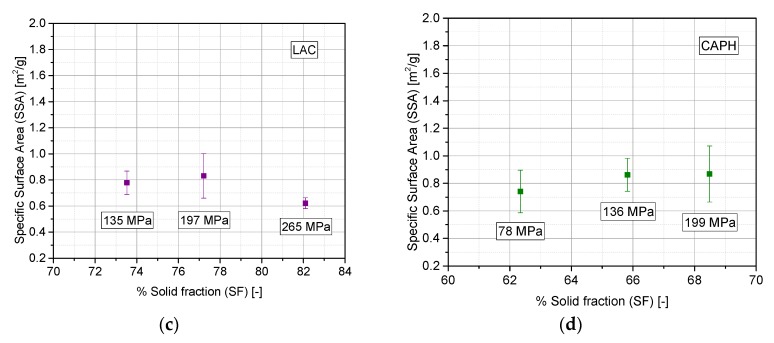
Surface area determination with Brunauer, Emmett, and Teller (BET) (*n* = 4, for each material and compaction pressure); (**a**) MCC-A; (**b**) MCC-P; (**c**) LAC; (**d**) CAPH.

In order to strengthen the derived hypothesis of the deformation mechanism, the evolution of the specific surface area was determined by BET (Figure 7), as also performed in the literature [9,41]. In a systematic study about the different types of specific surface area evolution with compaction pressure, at least three behaviors were reported [42]. (i) The first was an increase of the surface area at low pressure, which was linked to the initial fragmentation of particles. At a certain pressure, a maximum is achieved, and beyond this point, the specific surface area decreases because of particle recombination and pore closing. (ii) For materials that only exhibit plastic deformation without fragmentation, a decrease in the specific surface area is observed with increasing compaction pressure. (iii) For purely brittle materials, which densify by means of the continuous formation of new surfaces, the specific surface area steadily increases with compaction pressure. MCC-A/P can be described as purely elasto-plastic (profile ii). Our study shows that CAPH exhibits a general brittle behavior (iii), whereas LAC shows the transition reported in (i). This general trend of LAC, which includes a specific surface area local maximum at 197 MPa in our case, was also observed by Busignies et al. [43], and it was also reported for magnesium carbonate and phenacetin [44,45]. For a pressure lower than $P_{y}$, a clear fragmentation profile is identified; above this limit, particles’ particle contact areas tend to enlarge, closing pores, and therefore, the specific surface area decreases.

For ductile excipients, the physical reason for the described effects are the reduction and closing of interstitial pores by plastic deformation, and the enlargement of the particle–particle contact areas accounts for the reduction of the specific surface area. In contrast to that, in brittle powders, the ongoing fragmentation generates higher numbers of smaller particles that accommodate in the voids of larger particle packings, but still provide higher specific surface areas. The decrease in the specific surface area for LAC at very high solid fractions over 0.8 is tentatively explained by more densely packed particles that accordingly form more particle–particle contact areas, which do not contribute to the surface area assessed by gas adsorption. CAPH does not show this phenomenon (in the investigated range of compaction stresses), because lower solid fractions are reached. These general findings on the specific surface area progression are in agreement with the considerations presented in Section 3.2, namely, two distinguishable relaxation profiles for brittle and ductile powders. 

### 3.4. Sensitivity Analysis of Maximum Indentation Force and Structural Inhomogeneity

**Figure 8:**
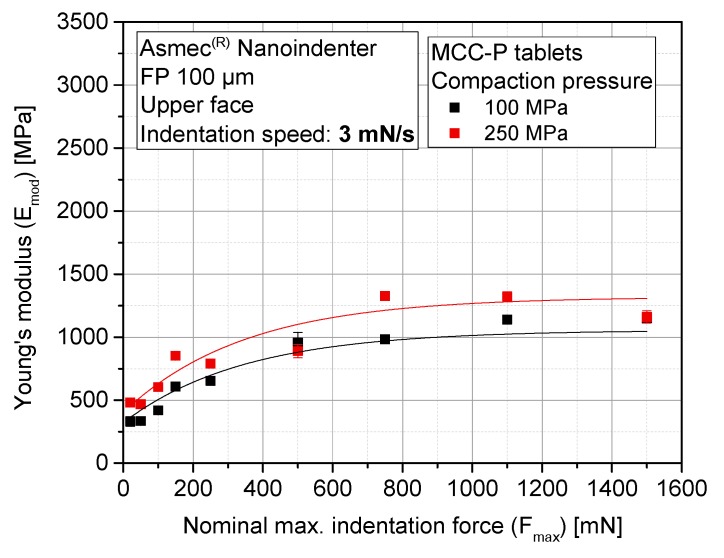
Median elastic modulus of MCC-P tablets as a function of the maximum indentation force for different nominal compaction pressures (*n* = 79 for each compaction pressure (CP) and indentation force).

**Figure 9:**
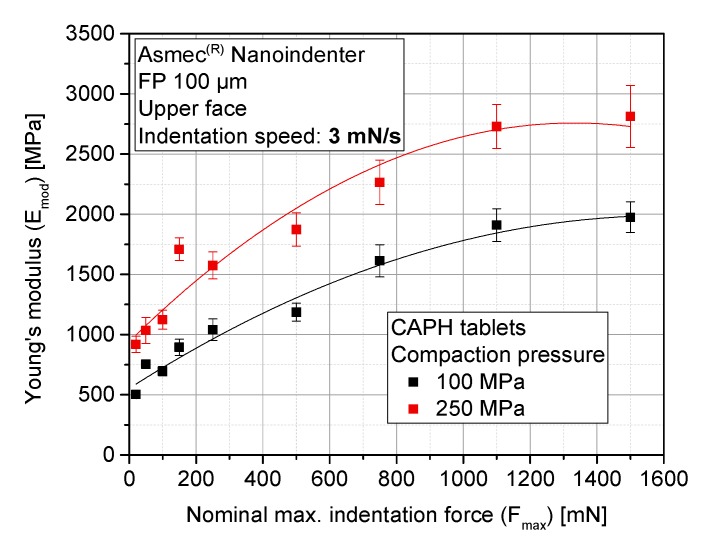
Median elastic modulus of CAPH tablets as a function of the maximum indentation force for different nominal compaction pressures (*n* = 79 for each CP and indentation force).

In order to find the adequate range for a meaningful and representative determination of $E_{mod}$, a study of the effect of maximum indentation force ($F_{max}$) was performed. Toward this goal, two representative materials with opposite elasto-plastic behavior were analyzed: Figure 8 provides the evolution of the elastic modulus of MCC-P tablets for two nominal compaction pressures (CP) at the tablet press (100 MPa and 250 MPa), whereas Figure 9 provides a similar analysis with CAPH tablets.

An increase in $E_{mod}$ with rising maximum indentation force has been determined regardless of the nominal CP. It is clearly shown that with higher tablet densification (a higher nominal CP), stiffer tablets are produced. The relative standard deviation of all of the tests remains constant for all of the indentation forces. $E_{mod}$ tends to converge to a constant value for maximum indentation forces above 1000 mN in the case of CAPH, and 750 mN in the case of MCC-P.

An explanation for this trend can be found in the internal structure of compacts. Porous tablets consist of a series of granules that suffer an additional local secondary compaction during indentation, as a consequence of grain boundary fracture and grain rearrangement [46]. These grain boundaries and the subsequent blocking of dislocations conveys a decrease of the relative grain size, resulting in an increase of hardening, and thus, stiffness. This hardening mechanism is more effective in a brittle material such as CAPH compared to MCC: the $E_{mod}$ of CAPH approximately duplicates from indentation forces of 250 mN to 1500 mN, whereas for MCC-P, $E_{mod}$ is roughly 1.5 times higher. At the same time, according to the particle sizes provided in Table 1, the indented area ranges from half to the entire area of one single primary particle (as the indentation depth is one magnitude lower than the indentation diameter), which makes the measured stiffness dependent on whether the indentation area is located between two adjacent particles or on top of one of them. This factor, which was briefly presented in the Introduction, can constitute an important source of experimental deviations.

**Figure 10:**
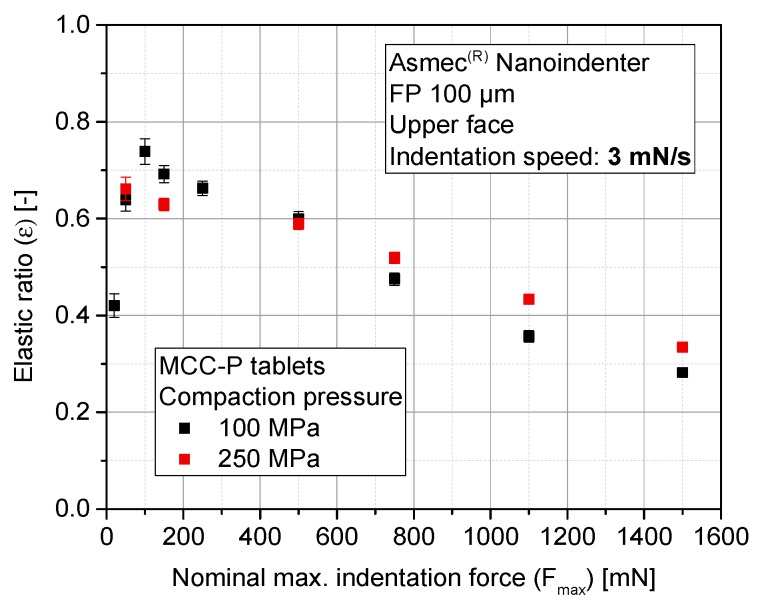
Elastic ratio (ε) of MCC-P tablets as a function of the maximum indentation force for different nominal compaction pressures.

**Figure 11:**
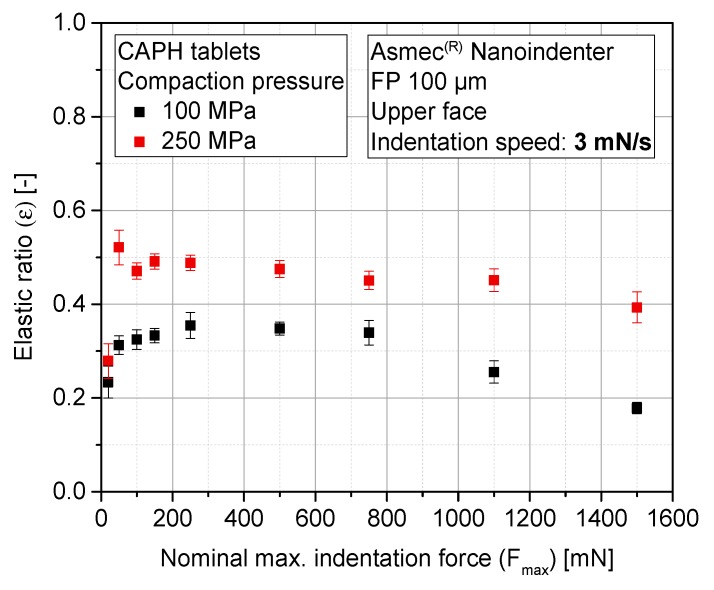
Elastic ratio (ε) of CAPH tablets as a function of the maximum indentation force for different nominal compaction pressures.

**Figure 12:**
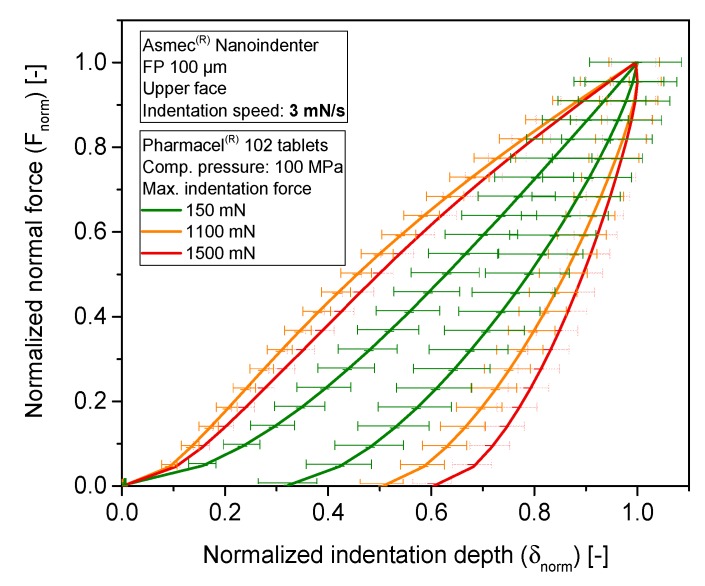
Normalized force–displacement indentation curves. Confidence intervals for *n* = 30 assuming normally distributed results for a 95% of probability. Material: MCC-P. Compaction pressure: 100 MPa.

Stiffness results are complemented with the elastic compression work ratio of the indentation tests (ε). It is defined as the ratio of the area below the unloading curve to the area below the loading curve. This parameter is examined for MCC-P (Figure 10) and CAPH tablets (Figure 11). The ε values of MCC-P and CAPH for the three highest indentation forces (750 mN, 1100 mN, and 1500 mN) are at least 10% lower for the CPs of 100 MPa as compared with corresponding value of 250 MPa. The resulting applied pressures during indentation (95 MPa, 140 MPa, and 191 MPa, respectively) are in the same range or higher than the nominal compaction pressure of the tableting process itself. As a result, a secondary, local compaction takes place, which is characterized by a high plastic deformation (loading curve) and a slower withdrawal of the punch during unloading due to a poor elastic recovery, and thus, a smaller measured $E_{mod}$. This effect can qualitatively be discerned with the profile differences between the indentation profiles at 150 mN, 1100 mN, and 1500 mN (Figure 12). As it can be seen, the shape of the loading part evolves from a concave to a convex curve above a critical indentation force, hinting at yielding events, and some incipient creeping is observed at the beginning of the unloading curve for higher indentation forces. Entering into this densification zone during compaction might have a parallel effect: localized particle rearrangement, elasto-plastic deformation, and in some indents, the reaching of pores, which increases the standard deviation of the results. Part of this tendency change can be attributed to the variable contact area with the indentation depth, as a consequence of the cut cone shape of the indenter. 

Finally, to a smaller extent, similar local compaction phenomena have been observed for CAPH, probably having its origin on the lower sensitivity of brittle materials toward ductile or viscous deformation, making them hard to be re-densified [27]. For CAPH as well as MCC, previously explained phenomena have a macroscopic translation to explain the inverse proportionality between ε and $F_{max}$. Derived from the stabilization of stiffness from 750 mN, by increasing the maximum indentation force, the plastic work applied on the sample surface (N∙m) rises in quadratic proportion. However, $E_{mod}$ stabilizes at the unloading part of the curve, and therefore, elastic work does not increase in the same proportion.

For the sake of simplicity and to avoid either the effects of adhesive forces between the tablet and the indenter (non-negligible at indentation depths below 1 mN) and the uncertainty region defined by the previously described local compaction (for the weakest tablets, 200 mN), an indentation force of 150 mN is set for the subsequent tests throughout the surface of the tablets.

### 3.5. Evolution of Stiffness with Compaction Pressure at Different Locations of the Tablet (Upper, Lower, and Lateral Faces)

**Figure 13:**
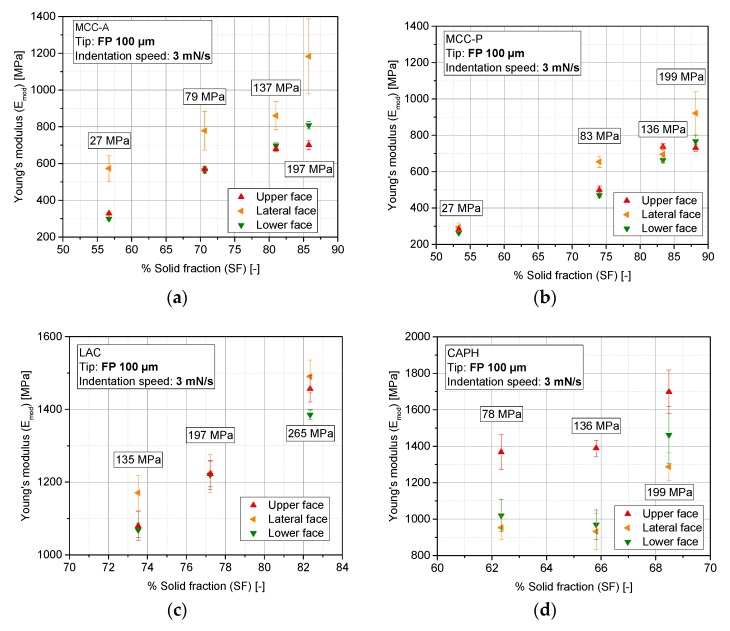
Elastic modulus as a function of the solid fraction of tablets for different excipients; (**a**) MCC-A; (**b**) MCC-P; (**c**) LAC; (**d**) CAPH. Sampling size: eight tablets per solid fraction (SF) and face, 164 indents at each tablet.

Figure 13 outlines the results of the indentation tests at the upper, lower, and lateral surfaces of the tablets. For each solid fraction and material, 79 different locations on the upper and lower surfaces and eight on the lateral wall of the tablet are considered. 

A tendency to a directly proportional correlation between SF and $E_{mod}$ was found for the upper and lower faces for MCC-A/P and LAC. For all three excipients, stiffness fluctuations between the upper and the lower faces of the tablet are scarce. A different behavior has been identified for CAPH (Figure 13d). A stiffness difference of around 30–40% between the top surface and all of the other parts of the tablet suggests a densification gradient from the upper part to the bottom. This is attributed to the friction forces occurring between the powder and the die wall, which is a common problem in uniaxial compaction that is traditionally solved by admixing a lubricant. Ellison et al. reported this effect for the uniaxial compaction of brittle powders employing a static lower punch and a movable upper punch [47]. For a lactose monohydrate grade, structural density differences were only relevant below a content of 0.25% (*w/w*) of magnesium stearate. Above this homogeneity threshold, which is the scenario of the current study (1% (*w/w*) of MgST), no drastic differences between the axial regions of the LAC tablets should be identified. This might not be the case for CAPH at 1% (*w/w*) of MgST, and the occurrence of a density gradient resulting from a low lubricant content is presumed.

Another outcome of this analysis is the stiffness difference between the axial and radial directions of the tablet; lateral faces are in most cases stiffer than the axial parts, which is a well-reported phenomenon in the literature [9,48]. This stiffness anisotropy is more pronounced in the case of ductile materials (MCC) than brittle materials. Nevertheless, this anisotropy does not have its origin in the primary shape of the granules, because, as reported in Section 3.1, they present an equiaxed morphology.

A possible explanation can be derived from the phenomenological description of compaction. Upon this process, the vertical dimension of the powder particles is reduced. The normal direction of compaction becomes a preferential direction for the contact formation. This circumstance has been experimentally validated by some authors with the appearance of the fracture surfaces and the numerical examination of the number of coordination and the fabric tensor evolution [49]. However, this geometrical consideration is not sufficient to account for the overall anisotropy of the system, especially the higher extent of it in ductile powders [4]. As presented before, the two mechanisms of energy dissipation taking place simultaneously after compaction are responsible for this effect: elastic recovery and Poisson’s effect. As a consequence of the latter, an asymmetrical elastic recovery after compaction provokes an axial expansion and a reduction of the radial stress within the tablet, while the outer dimension initially stays constant due to the confinement. For brittle excipients such as CAPH and LAC, a new contact formation due to the enlargement of contact areas between single particles caused by plastic deformation plays a minor role. In this case, the energy provided during compaction is transmitted and dissipated through the fragmentation of primary particles and the interlocking of the fines that are originated. Primary particle breakage is more likely to occur at the tablet lateral part than in the center and after relaxation. The structure is distinguishably more tensioned at the radial direction than at the axial. This phenomenon, combined with the MCC grades storing energy mainly with elastic deformation, which is more efficient and does not affect the structure integrity, explain the severe stiffness difference between LAC/CAPH and MCC.

In a study of the uniaxial compaction of SiO_2_ beads, which are a hard/brittle slightly cohesive material, Strege et al. studied the structural anisotropy from an in situ reconstruction of the contact alignment and arrangement by means of X-ray microtomography [50]. Beginning with an initial perfectly isotropic configuration, anisotropy increased with pressure, reaching a maximum value. Due to the continuous presence of friction, the anisotropy was meant to exist even for infinitive pressure.

Densification through fragmentation may also have an effect on strength anisotropy. As a matter of fact, the densification more effectively strengthens the side surface of MCC tablets in comparison with those made of brittle materials. When fragmentation occurs for plastically deforming materials such as MCC-A/P, the formation of fines is caused by diametrical fracturing, instead of chipping, lateral erosion. These fines rearrange directly after their generation, and tend to accommodate inside the interstices of larger particles, causing the overall densification of such materials.

### 3.6. Cumulative Property Analysis

**Figure 14:**
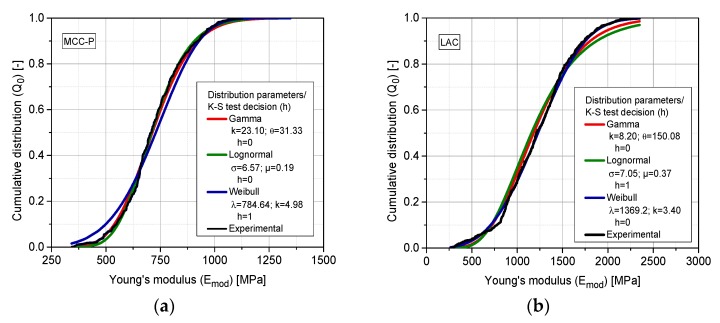
Fitted number cumulative distributions ($Q_{0}$); (**a**) MCC-P 138.0 MPa upper face; (**b**) LAC 197.0 MPa upper face. The legend summarizes the fitted parameters for each distribution and the result of the Kolmogorov–Smirnov (K–S) hypothesis test. Sampling size: eight tablets per SF and face, 79 indents at each tablet.

Due to the local distribution of the indentation points, it is meaningful to integrate the test outcome with a number-based cumulative distribution ($Q_{0}$). Toward this goal and prior to the fitting of one particular distribution, a one-sample Kolmogorov–Smirnov test (K–S test) is executed for each sample [51,52]. The result of this test returns the decision for the null hypothesis that the experimental data comes from a standard distribution, against the alternative that it does not come from such a distribution. The result h is 1 if the test rejects the null hypothesis at the 5% significance level, or 0 in the opposite case. According to the shape of the cumulative experimental data of $E_{mod}$, a range of skewed distributions (gamma, log-normal, and Weibull) are suggested. Figure 14 provides two examples: (**a**) MCC-P 138.0 MPa with measurements at the upper face; and (**b**) LAC 197.0 MPa also at the same tablet face.

As shown, the experimental data of $E_{mod}$ can only be characterized with a good agreement with skewed distributions. Regardless of the excipient, compaction pressure, and face, the so-called gamma two-parameter distribution is the only one that is capable of accounting for the kurtosis of the distributions as assessed by the K–S hypothesis test. The probability density function of gamma distribution is defined as:

(8) f(x)=1Γ(k)θkxk−1e−xθ,

where k represents the shape parameter and θ represents the scale parameter. Γ(k) is the gamma function represented by:

(9) $$\Gamma \left(k\right)=\int_{0}^{\infty }x^{k-1}e^{-x}dx,$$

**Figure 15:**
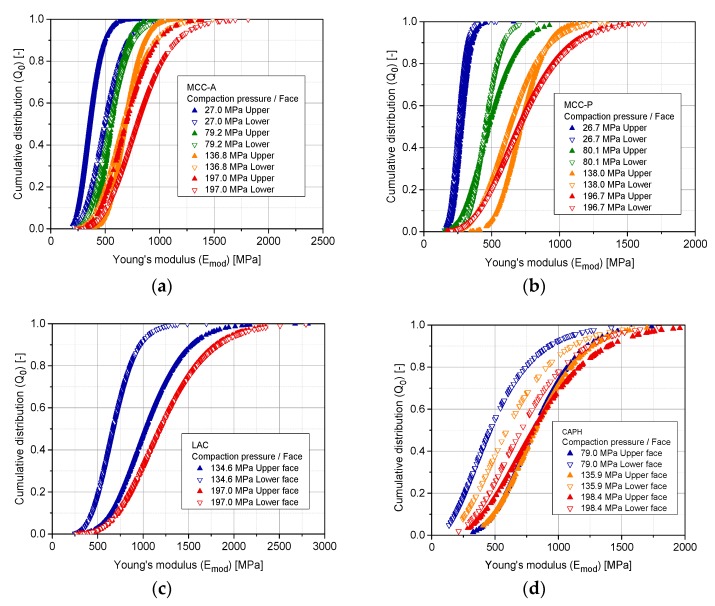
Fitted volumetric/weight cumulative gamma distributions ($Q_{0}$) as a function of the maximum compaction pressure for the upper and lower tablet faces of the following excipients; (**a**) MCC-A; (**b**) MCC-P; (**c**) LAC; (**d**) CAPH. Sampling size: eight tablets per SF and face, 79 indents at each tablet.

According to the parametrization of a gamma distribution, the larger the scale parameter (θ), the more spread out the distribution, whereas an increase in k shifts the distribution toward larger values. For the upcoming representation, the k and θ of the gamma distribution are fitted for each test case using the *fitdist* probability distribution fitting tool of MATLAB^TM^ (Table 2). Figure 15 accounts for the representation of the fitted cumulative gamma distributions for $E_{mod}$. The scale factor θ increases with the increasing compaction pressure for all of the analyzed materials, clearly indicating the wider dispersion of stiffness at the highest degree of compaction. These results contradict the notion that the higher the degree of densification of the structure, the more compact and thus, more homogeneous it should be [18]. However, this effect may have its origin in the accumulation of effects ruling different stages of compaction: rearrangement, elasto-plastic deformation for ductile materials, fragmentation, and dislocations interlocking for brittle materials. This complex interplay of stress events, with some of them predominant through successive stages of compaction, leads to an increasingly heterogeneous structure with compaction pressure. Additionally, at the border, compaction is hindered because the contact formation is reduced and the elastic expansion after ejection from the die yields the highest stresses perpendicular to the radial direction on the circumference of the tablet.

### 3.7. Property Distribution over Tablet Faces

**Figure 16:**
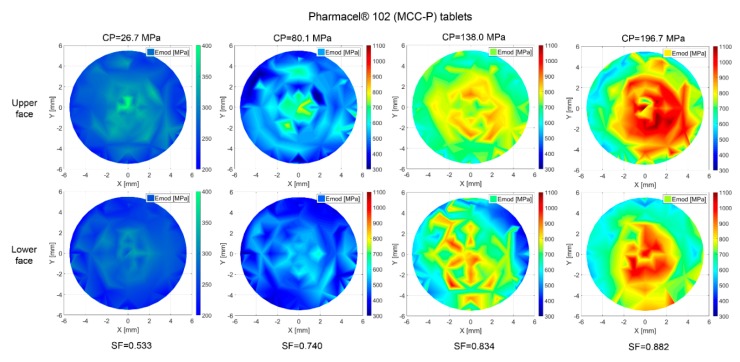
$E_{mod}$ distribution over tablet surface for MCC-P tablets. Coordinates [0, −5.625] correspond to the frontal part during compaction. The filling shoe approaches from [−5.625, 0] with a rotational cycle, and it withdraws following its same path to [5.6250, 0]. Sampling: eight tablets per pressure and face; 79 indents at each tablet face.

**Figure 17:**
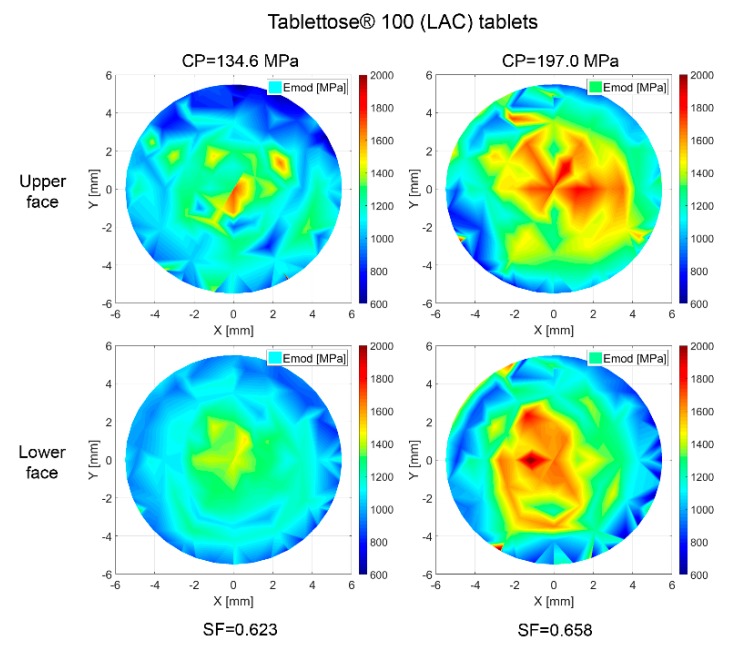
$E_{mod}$ distribution throughout the tablet surface for LAC tablets. Coordinates [0, −5.625] correspond to the frontal part during compaction. The filling shoe approaches from [−5.625, 0] with a rotational cycle and it withdraws following its same path to [5.6250, 0]. Sampling: eight tablets per pressure and face; 79 indents at each tablet face.

The scattered data of Section 3.2 is processed with a Delaunay triangulation in MATLAB^®^ in order to obtain the stiffness distribution at the surface of the compacts [53]. The results of this analysis are displayed in representative color diagrams for MCC-P (Figure 16) and LAC (Figure 17) tablets. 

An increase in stiffness values of at least 100% between the edges and the central part of the upper and lower faces has been determined regardless of the compaction pressure, face, and excipient material. This phenomenon implies that the mid of the tablet has a higher degree of densification, giving hint at an also higher number of contacts. Ellison et al. reported just the opposite case for poorly lubricated formulations. The local stress resulting from friction at the space between the powder and the die results in an acute-angled wall resistance to powder motion [47]. In the current scenario, it is suggested that the driving force in densification lays on geometrical considerations, namely, higher contact formation likelihood at the center of the tablet [54]. In the same line, out-of-die elastic relaxation, as described in Section 3.3, is more plausible at radial locations due to lower geometrical resistance. 

Han et al. analyzed the stress and density distribution throughout different stages of uniaxial compaction of cylindrical tablets of Avicel^®^ PH 101 based on a continuum modeling via FEM by applying the modified Drucker–Prager Cap model, keeping the position of the lower punch constant and exerting pressure with the upper punch [55]. Considering a frictional term on the formulation of the model, during compression, decompression, and ejection, stress distributions were irregular, causing a non-uniform density distribution. As a result of that, the top and the bottom corners were the most and the least densified regions, respectively, with each of them having a peak difference to the overall average density of the tablet of around 12%. Not identifying this highly densified region at the top corner of the tablet in Figure 16 and Figure 17 is plausible because of the symmetrical movement of the punches for the current analysis. However, a reduced sampling of the adjacent regions of the edges due to the impossibility of satisfactorily performing an indent from a relative radial distance to the center of 0.95 (560 µm) may not allow in any case identifying an overall lateral hardening. 

The omnipresent $E_{mod}$ gradient between the upper and the lower faces for all of the compaction pressures and excipient materials in Figure 16 and Figure 17 constitutes a phenomenon that is also observed by Eiliazadeh et al. [56]. In this study, for Avicel^®^ PH 102 and cylindrical instrumentation, they traced the density distribution by admixing steel balls to the formulation, which permitted the density reconstruction by X-ray imagery. They identified regions of high density in contact with the top punch and a vector force acting outwards with respect to the loading axis. A frictional force at the die wall impedes the powder movement, and as a result, an uneven pressure distribution is generated. Toward the lower part of the tablet, particles experience less compressive force, which explains the lower density. 

**Figure 18:**
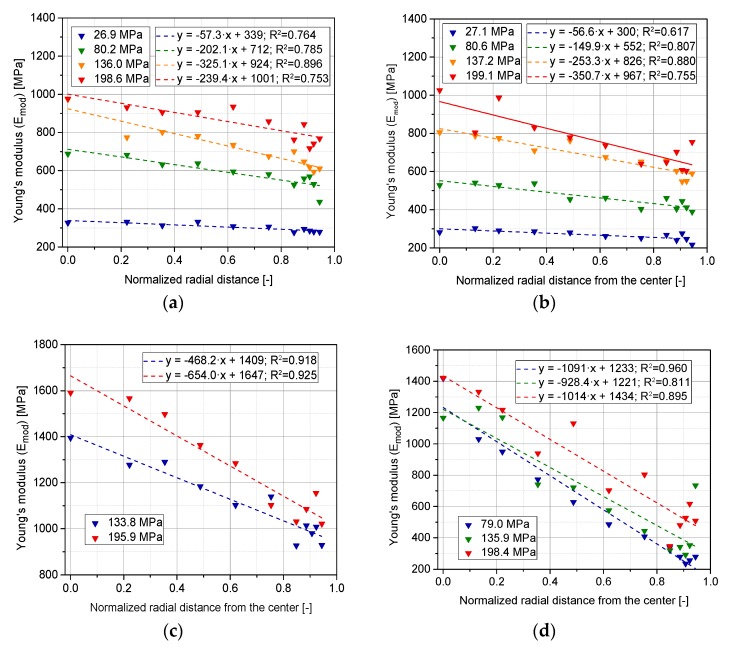
Radial evolution of $E_{mod}$ at the lower surface of the tablet for the following materials; (**a**) MCC-A; (**b**) MCC-P; (**c**) LAC; (**d**) CAPH. Sampling: eight tablets per SF and face, six indents at each radial distance.

A formal expression that assesses the dependence of stiffness on the normalized radius is presented in Figure 18. Linear fitted equations show a moderate agreement with the experimental data. It was previously reported that the elasticity of tablets composed of elasto-plastic materials is higher at the center, where the material is work-hardened and therefore able to resist further deformation [18]. Compressed particles that are situated at the center of the tablet are harder and deform plastically with lower elastic recovery. The extent of shearing at the adjacent regions with the die results in softer particles that elastically recover from deformation.

Experimental results also show a more pronounced decrease in $E_{mod}$ for both predominantly brittle materials (LAC and CAPH) for which densification mainly evolves by fragmentation. This includes two possible mechanisms: (a) fracturing at the contact points that generates debris, and (b) diametrical fracturing of particles due to secondary tensile stresses [9,57]. At the same time, particles with a low coordination number are more likely to contribute to the development of effective tensile stresses that causes fracture due to the lack of confinement and, by that, stress distribution to neighboring particles. Consequently, the tendency of fragmentation increases at the initial stages of compaction; it reaches a peak and then drops again due to an increase in the coordination number. For mechanism (a), particles require a rotation of the particle fragments to adjust the motion of adjoining particles. This phenomenon is more likely at the edges of the tablet, due to the effect of shear forces. According to the equation of Rumpf, the higher amount of fines generated at the edges would convey a stronger structure and therefore a larger $E_{mod}$ [58]. However, in the current scenario, a clear dominance of elastic relaxation has been assessed as the source of a lower density after relaxation, and therefore a lower $E_{mod}$.

## 4. Conclusions

The applicability of microindentation as a tool to describe the superficial heterogeneity of cylindrical pharmaceutical tablets with commonly used excipients has been proved. A planar, frustum-shaped indenter tip was used to map the stiffness ($E_{mod}$) (statistical and spatial) distribution alongside the upper, lower, and lateral faces. In-die compaction data is a valuable tool for examining deformation profiles and elastic recovery.

The results of the local stiffness distribution over tablet faces depicted a linear and directly proportional tendency between the solid fraction and $E_{mod}$ for the upper and lower faces, as well as remarkable stiffness anisotropy between the axial and radial directions of compaction. The highest extent of the stiffness anisotropy that was found for both ductile grades (MCC) in comparison with brittle powders (LAC and CAPH) has been attributed to the dual phenomena of overall elastic recovery and Poisson’s effect in the relaxation kinetics. As a reinforcement of this analysis, the evolution of the specific surface area elucidated the respective densification mechanism and its implementations toward anisotropy. For ductile excipients, the increase in contact surface area as well as the reduction and closing of interstitial pores explains the reduction of surface area with increasing compaction pressure. For brittle powders, densification evolves through fragmentation and the subsequent filling of voids.

Gamma two-parameter cumulative distribution provides the best kurtosis depiction of the measured distributions. Scale factors, and through that the width of the distribution, remarkably increase toward higher compaction pressures. Finally, local stiffness distributions throughout the tablet surfaces and their radial dependence depicted an increase of stiffness of 100% from the edge to the center of the tablets. This phenomenon means that the mid of the tablet has a higher degree of densification, and also a higher absolute number of contacts, and in the same line, the likelihood of elastic relaxation is more plausible at radial locations due to lower geometrical impedance. The occurrence of this structural gradient might have significant implications on the damaging likelihood, especially by chipping, throughout finishing by coating. Good practices by tableting, for instance by an adequate tablet lubrication or an extended residence time at the press, contribute to reduce the overall anisotropy and enhance the product quality, but they might not be sufficient.

## Figures and Tables

**Table 1:** Physical properties of the powders of interest: particle size distribution (PSD) from volumetric/mass cumulative distributions and true/skeletal density.

Material	x_10_ (Q_3_) (µm)	x_50_ (Q_3_) (µm)	x_90_ (Q_3_) (µm)	Span (-)	Measuring Technique	True Density (kg·m^−3^)	Measuring Technique
MCC-A	82.9	224.6	379.3	1.32	Dynamic particle image analysis	1541.1	Helium pycnometry
MCC-P	28.3	86.5	173.8	1.68		1533.7	
CAPH	131.6	210.5	298.4	0.79		1763.3	
LAC	34.3	107.8	302.0	2.48	Laser diffraction	2766.0	

**Table 2:** Fitting volumetric/weight cumulative parameters for gamma distributions ($Q_{0}$) as a function of the maximum compaction pressure for the upper and lower tablet faces.

Material	Compaction Pressure (MPa)	Face	Gamma k Factor (-)	Gamma θ Factor (-)
MCC-A	27.0	Upper	11.20	29.29
		Lower	13.10	42.95
	79.2	Upper	24.15	23.41
		Lower	24.15	23.41
	136.8	Upper	27.27	25.36
		Lower	10.77	64.40
	197.0	Upper	11.40	62.24
		Lower	10.32	79.97
MCC-P	26.7	Upper	24.75	11.34
		Lower	25.98	10.02
	80.1	Upper	8.43	59.07
		Lower	22.17	20.86
	138.0	Upper	23.10	31.33
		Lower	9.78	67.52
	196.7	Upper	6.90	105.84
		Lower	6.90	108.56
LAC	134.6	Upper	8.98	118.56
		Lower	10.77	64.40
	197.0	Upper	8.20	150.08
		Lower	7.81	159.26
CAPH	79.0	Upper	8.09	103.87
		Lower	2.95	176.51
	135.9	Upper	7.61	112.02
		Lower	3.70	176.57
	198.4	Upper	4.59	187.66
		Lower	4.56	167.26
